# The Oral–Gut–Systemic Axis: Emerging Insights into Periodontitis, Microbiota Dysbiosis, and Systemic Disease Interplay

**DOI:** 10.3390/diagnostics15212784

**Published:** 2025-11-03

**Authors:** Amani M. Harrandah

**Affiliations:** Department of Basic Oral and Clinical Sciences, Umm AlQura University College of Dental Medicine, Makkah P.O. Box 715, Saudi Arabia; amharrandah@uqu.edu.sa

**Keywords:** oral-gut-dysbiosis, periodontal bacteria, oral microbiota, gut dysbiosis, systemic diseases, periodontitis

## Abstract

The oral cavity harbors one of the most diverse microbial ecosystems in the human body, second only to the gut. Periodontitis, a chronic inflammatory disease arising from oral microbiota dysbiosis, has been increasingly associated with systemic disorders such as diabetes mellitus, atherosclerosis, rheumatoid arthritis, inflammatory bowel disease, and neurodegenerative conditions. Although hematogenous dissemination of oral pathogens and inflammatory mediators has long been proposed as a mechanistic link, emerging evidence identifies the oral–gut axis as a novel bidirectional pathway. Swallowed oral pathobionts, such as *Porphyromonas gingivalis* and *Fusobacterium nucleatum*, can colonize the gut, disrupt the intestinal barrier, and induce dysbiosis, immune imbalance, and metabolic alterations that aggravate systemic inflammation and disease progression. In contrast, gut dysbiosis, especially in obesity or high-fat-diet models, can exacerbate periodontal tissue destruction through hyperuricemia, altered bone metabolism, and Th17/Treg immune imbalance. Experimental and clinical studies further support this reciprocal relationship, implicating microbial, metabolic, and immune crosstalk in both oral and systemic pathology. Understanding this oral–gut–systemic axis offers a paradigm shift in diagnostics and therapeutics, focusing on precision interventions such as microbiome modulation, probiotics, and integrated oral care to mitigate systemic inflammatory burden and improve overall health outcomes.

## 1. Introduction

The oral cavity, the initial site of the digestive tract, hosts one of the most diverse microbial ecosystems in the human body, second only to the gut [1]. This community plays a crucial role in maintaining mucosal and systemic homeostasis [2]. Recently, the term “oralome” has been introduced to comprehensively describe the dynamic and multidimensional interactions between the oral microbiome and its host, encompassing microbial, molecular, and immunological cross-talk that collectively influence oral and systemic health [3]. Periodontitis is a chronic inflammatory disease caused by oral microbiosis, which disrupts immune balance and barrier integrity [4]. Periodontitis arises from a shift in the oral microbiome from a balanced (eubiotic) to a dysbiotic state, progressing from early “plaque-based” theories to modern ecological and polymicrobial models that emphasize microbial interactions and host response. The current Polymicrobial Synergy and Dysbiosis model recognizes periodontitis as a community-driven disease that involves various bacterial, fungal and viral species that collectively sustain chronic inflammation and tissue destruction [3]. Epidemiological evidence has suggested an association between periodontitis and multiple systemic diseases such as cardiovascular disease, diabetes mellitus, rheumatoid arthritis, Inflammatory Bowel Disease (IBD), and even neurodegenerative disorders [4,5,6].

Traditionally, multiple lines of evidence, such as the detection of periodontal bacteria’s DNA after periodontal treatment, suggested that the hematogenous spread of oral bacteria occurs through inflamed tissues and pro-inflammatory mediators from inflamed periodontal tissues. However, the number of oral bacteria detected in the bloodstream differs greatly depending on the testing approach and the individual’s oral condition, and a unified conclusion has not yet been reached [4,5,6].

Furthermore, recent advances in sequencing technologies and microbiome research have shed light on an alternative pathway that links oral and systemic health: the oral–gut axis. In this model, oral pathobionts such as *Porphyromonas gingivalis* (*P. gingivalis*) and *Fusobacterium nucleatum* (*F. nucleatum*) are continuously swallowed with saliva and can survive gastric acidity to reach the intestinal tract [7,8,9]. Once established, these microorganisms may disrupt the balance of the gut microbiota, leading to dysbiosis characterized by reduced microbial diversity, altered metabolite production, and impaired epithelial barrier function [9,10,11]. Such changes facilitate systemic dissemination of bacterial products, promote endotoxemia, and shift host immune responses, particularly through activation of Th17 pathways and suppression of regulatory T cells [12,13].

This oral–gut axis not only provides a mechanistic explanation for the observed association between periodontitis and conditions such as diabetes, atherosclerosis, and IBD, but also highlights the bidirectional nature of this relationship, where intestinal dysbiosis can in turn exacerbate periodontal inflammation. As a result, the oral–gut connection represents a paradigm shift in understanding the systemic impact of oral diseases and has emerged as a promising focus for both mechanistic studies and translational therapeutic strategies.

This review will discuss recent reports on the interaction between the oral and intestinal microbiota and the causes of systemic pathological changes, including periodontal tissue.

## 2. Oral Dysbiosis, Gut Colonization, and Their Interplay in Systemic Diseases

A substantial body of evidence indicates that oral pathogens can survive transit through the gastrointestinal tract and subsequently colonize the gut, a phenomenon particularly prominent in individuals with compromised intestinal microbiota. The oral cavity, especially in the context of severe periodontitis, serves as a significant reservoir for this microbial translocation, with patients’ ingestion of a continuous high-volume bolus of oral bacteria. Estimates suggest that patients with periodontitis may swallow as many as \(10^ {910^ {10}\) bacterial cells daily, a massive microbial load that challenges the gut’s normal “colonization resistance”. This phenomenon has been mechanistically validated in animal models, where oral administration of pathogens such as *P. gingivalis* has been shown to result in their successful persistence within the gastrointestinal tract. Intestinal colonization by these oral pathobionts subsequently alters the composition of the native gut microbiota, disrupts the integrity of the intestinal barrier, and contributes to a systemic inflammatory state. This evidence highlights a dynamic and critical oral–gut axis in which oral pathogens exploit a disrupted gut microenvironment to influence systemic health [9,14,15,16,17].

Among these translocating species, *F. nucleatum* has been one of the most consistently associated with gastrointestinal pathology. It has been frequently detected in colorectal carcinoma tissue and fecal samples from patients with colorectal cancer. Mechanically, *F. nucleatum* expresses FadA adhesin, which binds to E-cadherin in colonic epithelial cells, activating β-catenin signaling pathways that drive tumor cell proliferation. In addition, its Fap2 protein enables immune evasion by binding to the inhibitory receptor TIGIT on natural killer cells and T cells, thus dampening anti-tumor immunity. Beyond cancer, *F. nucleatum* has been implicated in exacerbating colitis by inducing pro-inflammatory cytokines such as IL-17 and TNF-α, further supporting its pathogenic role in the oral–gut axis (Table 1) [14,15,16,17,18].

Similarly, *P. gingivalis* has been identified as a key modulator of gut physiology. Experimental oral inoculation of mice with *P. gingivalis* results in decreased expression of tight junction proteins such as Tjp1 (ZO-1) and occludin (Ocln) in the intestinal epithelium, thus altering intestinal barrier function and leading to systemic endotoxemia [19]. Moreover, Oral administration of *P. gingivalis* has been shown to alter the gut microbiota, resulting in entero-hepatic metabolic disturbances that exacerbate hyperglycemia in obese murine models of type 2 diabetes [20]. Neurodegenerative models have demonstrated that *oral P. gingivalis* exacerbates Parkinsonian pathology and induces cognitive impairment through gut permeability disruption and neuroinflammation (Table 1) [21,22].

The systemic impact of oral–gut dysbiosis is extensive. In cardiovascular disease, metabolites such as trimethylamineN-oxide (TMAO) generated by the dysbiotic gut microbiota accelerate atherosclerosis [23]. Oral commensal *Klebsiella* spp., which is usually harmless in the oral cavity, can act as gut pathobionts when intestinal conditions are permissive. In murine models, colonization with Klebsiella pneumoniae induces strong Th1 responses, leading to colitis and systemic immune activation [24]. Furthermore, IBD is aggravated by oral bacteria colonizing the gut, which induce Th17-mediated immune responses. For example, ectopic gut colonization by Haemophilus parainfluenzae has been associated with IBD (Table 1) [25].

Interestingly, certain strains of Streptococcus mutans are a potential risk factor for aggravating ulcerative colitis (UC). This aggravation occurs when virulent oral strains, specifically those expressing a collagen-binding protein (CBP) and belonging to minor serotypes such as k or f, enter the bloodstream, often after dental procedures. These specific bacteria can resist phagocytosis and travel through the blood to the liver, where they are taken up by hepatocytes. The infection then stimulates the liver to produce the inflammatory cytokine interferon-gamma, which travels to the colon and intensifies existing inflammation. This blood-borne mechanism is significant, as oral administration of the bacteria does not produce the same effect, and patients with UC show a higher detection frequency of these specific, virulent S. mutans strains. Furthermore, Research has revealed a strong association between collagen-binding protein (Cnm) produced by certain Streptococcus mutans strains and the aggravation of hemorrhagic stroke. A 2011 Nature Communications study demonstrated that Cnm-positive serotype k strains can enter the bloodstream, adhere to exposed collagen in cerebral vessels, and disrupt platelet aggregation, thereby worsening intracerebral hemorrhage. The fact that recombinant Cnm protein alone induces hemorrhagic aggravation highlights its direct pathogenic role (Table 1) [26,27].

Furthermore, a group of bacterial species possessing genes that encode the enzymes β-glucuronidase and β-galactosidase, which are responsible for metabolizing conjugated estrogens, is collectively known as the “estrobolome.” Growing research interest has focused on this concept due to its potential role in influencing various diseases, including oral cancer. Although estrobolome-associated bacteria are predominantly found within the gut microbiota, emerging experimental evidence indicates a crosstalk between the oral and gut microbiomes. Notably, several oral bacterial species are also prevalent in the gut, where they may contribute to the activation of the estrobolome [28].

Collectively, these findings highlight the capacity of oral microorganisms not only to survive gastrointestinal passage but also to establish themselves as influential drivers of intestinal and systemic disease.

## 3. Experimental Models Used to Study Oral–Gut–Systemic Axis

**Figure 1:**
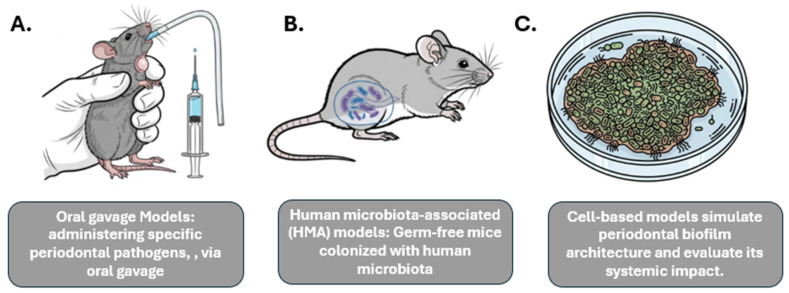
Experimental Models Used to Study Oral–Gut–Systemic Axis. This figure illustrates diverse experimental models utilized to explore the intricate connections within the oral–gut–systemic axis. (**A**) Oral gavage Models depict the administration of specific periodontal pathogens via oral gavage in an animal model (e.g., mouse) to study their systemic impact. (**B**) Human microbiota-associated (HMA) models involve germ-free mice colonized with human microbiota to investigate host-microbiome interactions and their systemic effects. (**C**) Cell-based models demonstrate in vitro systems simulating periodontal biofilm architecture and evaluating its systemic implications through analysis of secreted factors or interactions with host cells. These models provide valuable tools for understanding the complex interplay between oral health, gut microbiota, and systemic conditions.

To study the complex relationships within the oral–gut–systemic axis, researchers employ a variety of experimental models, most commonly using mice. These models allow for the manipulation of the oral microbiome and the observation of systemic health outcomes in a controlled setting (Figure 1).

Oral gavage models: This method involves administering specific periodontal pathogens, such as *P. gingivalis*, via oral gavage to observe their systemic effects. This approach has been shown to induce gut barrier disruption, lead to metabolic endotoxemia (the presence of LPS in the bloodstream), and elevate systemic pro-inflammatory cytokines [29].

Human microbiota-associated (HMA) models: This approach uses germ-free mice that are colonized with human microbiota, allowing for the study of the effects of a human-relevant microbiome. Studies have shown that mice receiving salivary microbiota from periodontitis patients develop systemic pathologies, such as hepatic steatosis, adipose inflammation, and worsened NAFLD and colitis [19].

In addition to animal models, in vitro models are used to investigate specific mechanisms, such as those related to inflammatory signaling pathways. For example, cell-based models can simulate periodontal biofilm architecture and evaluate its systemic impact.

While useful for high-throughput screening, a key limitation is their inability to fully replicate the complexity of the oral–gut–systemic axis [30].

## 4. Immunological Mechanisms

The oral–gut immune axis represents a critical bidirectional inflammatory pathway, where the primary immunological mediators are often Th17 cells. During active oral pathologies like periodontitis, oral pathobionts such as Klebsiella, Enterobacter, and *P. gingivalis* expand and are ingested. These bacteria translocate to the gut where they activate the inflammasome within lamina propria macrophages. Concurrently, periodontal inflammation generates oral pathobiont-reactive Th17 effector cells in the oral cavity that are imprinted with gut-specific homing markers. Upon reaching the intestine, these oral-origin Th17 cells are reactivated by the translocated oral pathobionts and amplify the gut’s inflammatory response, driving the onset and progression of colitis. Conversely, gut-primed Th17 cells can also migrate to the oral mucosa and exacerbate periodontal inflammation, with studies showing that ectopic gut colonization by oral pathogens like *P. gingivalis* can enhance Th17 differentiation in Peyer’s patches, with these cells subsequently accumulating in the mouth and worsening periodontitis. This highlights how localized dysbiosis in one mucosal site can trigger systemic immune responses that fuel inflammation in the other, establishing a dynamic and reciprocal inflammatory cycle [31,32,33,34].

Beyond the Th17-centric T-cell migration, other immune mechanisms contribute to the complexities of the oral–gut axis. An imbalance between pro-inflammatory Th17 cells and anti-inflammatory regulatory T cells (Tregs) is a key feature, as the gut microbiota and its metabolites, such as short-chain fatty acids, are known to shape the Th17/Treg balance. Oral bacteria can also be transported to the gut by invading and utilizing immune cells like macrophages and dendritic cells as “Trojan horses”. This mechanism bypasses digestive barriers, delivering pathogens directly to the intestinal immune system where they can influence macrophage polarization. For example, specific oral pathobionts can activate pro-inflammatory M1 macrophages and drive inflammation. Furthermore, the oral–bone–gut axis illustrates how oral inflammation leaves a lasting mark on the immune system, specifically on hematopoietic progenitor cells in the bone marrow. Oral infection can program these progenitors to produce a more inflammatory phenotype of immune cells, retaining an “inflammatory memory” that can circulate and contribute to systemic inflammation, including in the gut, even after the initial oral infection has subsided. These diverse pathways reveal a multi-layered, interconnected system where oral health profoundly impacts systemic immune function [35,36,37,38,39].

## 5. Bidirectional Influence

**Figure 2:**
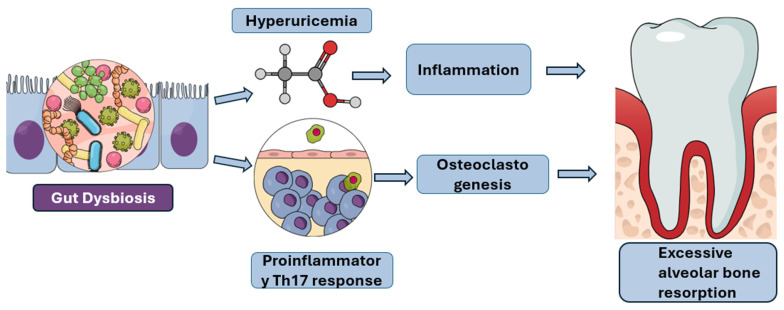
Pathways Linking Gut Dysbiosis to Excessive Alveolar Bone Resorption: This figure illustrates the proposed mechanisms by which gut dysbiosis contributes to excessive alveolar bone resorption, a hallmark of periodontal disease. Gut dysbiosis can lead to hyperuricemia, which in turn promotes inflammation. Concurrently, gut dysbiosis can also induce a proinflammatory Th17 response and stimulate osteoclastogenesis. These interconnected pathways ultimately culminate in the accelerated breakdown of alveolar bone.Furthermore, gut dysbiosis shifts the immune landscape by promoting pro-inflammatory Th17 responses at the expense of protective regulatory T cells (Tregs), thereby inducing Th17-driven osteoclastogenesis and bone loss. When this Th17/Treg imbalance occurs in the gut due to dysbiosis, the pro-inflammatory immune cells are primed to migrate to distant sites, including the oral cavity, where they worsen local inflammation and accelerate periodontal bone destruction [Figure 2] [47]. In addition to Th17 activation, obesity and metabolic dysfunction contribute to systemic inflammation through adipose tissue-derived inflammatory mediators, which perpetuates a vicious cycle. This cycle links gut dysbiosis to heightened systemic inflammation, which in turn impairs the periodontium’s ability to resist bacterial challenge, ultimately accelerating periodontal disease and bone loss [48].

Emerging evidence indicates that the interaction between the oral and gut microbiota is bidirectional. While oral microorganisms can translocate to the gastrointestinal tract and contribute to gut dysbiosis and systemic pathologies, alterations in the gut microbiome have likewise been shown to modulate the immune response, metabolic status, and microbial ecology of the oral cavity, thereby influencing the onset and progression of oral diseases. For example, dysbiosis within the gut exacerbates periodontal inflammation by disrupting the host’s systemic metabolic and immune balance, particularly through diet-induced changes. Furthermore, the close association between periodontitis and IBD is evidenced by the increased prevalence of IBD among patients with periodontitis and the greater incidence and severity of periodontitis in individuals with IBD. This bidirectional relationship suggests that periodontitis and IBD are interconnected rather than independent entities, forming an oral–gut axis through which each condition exacerbates the other, thereby establishing a self-perpetuating pathogenic cycle [40]. In high-fat-diet models, an altered gut microbiota leads to elevated levels of serum uric acid, a key metabolite derived from the purine degradation pathway. This systemic hyperuricemia promotes inflammation and drives excessive alveolar bone resorption, demonstrating a direct mechanistic link between diet-induced gut dysbiosis and periodontal tissue destruction [Figure 2] [40,41]. Conversely, a healthy gut microbiota contributes to bone and systemic health through the production of short-chain fatty acids (SCFAs), which play a pivotal role in maintaining bone homeostasis and intestinal integrity. SCFAs such as butyrate and propionate promote osteoblast activity, inhibit osteoclast differentiation, and enhance bone mineral density by modulating immune cell differentiation and systemic metabolism. Beyond their skeletal benefits, SCFAs—particularly butyrate—serve as the primary energy source for colonocytes, the epithelial cells lining the colon [42]. A reduction in SCFA-producing bacteria leads to diminished SCFA levels, resulting in impaired gut barrier function. This weakening of the intestinal epithelium increases permeability, a condition commonly referred to as “leaky gut,” which facilitates the translocation of bacterial endotoxins such as lipopolysaccharide (LPS) into the systemic circulation. The resulting endotoxemia triggers chronic, low-grade inflammation that has been strongly implicated in the onset and progression of IBD, colorectal cancer, and other systemic inflammatory disorders. Beyond the gut, low SCFA levels can have detrimental effects on metabolic and immunological health. Acetate and propionate are SCFAs that regulate glucose and lipid metabolism in tissues like the liver and fat cells. A decrease in their production can contribute to metabolic disorders, including obesity, insulin resistance, and type 2 diabetes. Furthermore, SCFAs influence the gut–brain axis and immune system regulation. Specifically, butyrate promotes the development of regulatory T cells, which help to suppress excessive immune responses and prevent autoimmunity. Without sufficient SCFAs, this critical immunomodulatory function is impaired, leading to a state of chronic inflammation that can affect multiple systems throughout the body [43,44,45,46].

The intricate interplay of these microbial, metabolic, and immunological pathways reveals how gut health profoundly influences periodontal disease progression, offering a potential target for therapeutic intervention through managing obesity and metabolic disorders.

## 6. Clinical Implications

The oral–gut axis represents a critical frontier in understanding systemic health, reflecting a bidirectional communication network where microbial homeostasis is pivotal to preventing disease. Oral pathogens, such as *P. gingivalis* and *F. nucleatum*, can translocate to the gut, disrupting the delicate intestinal microbiome and exacerbating systemic inflammation, which is implicated in metabolic and autoimmune pathologies. This dysbiotic crosstalk can be harnessed for novel diagnostic and therapeutic strategies. For diagnostics, the analysis of salivary and fecal microbiota offers a non-invasive approach to identifying predictive biomarkers for diseases like colorectal cancer and IBD, reflecting systemic changes. Therapeutically, interventions aim to restore microbial balance; these include the use of prebiotics and probiotics, fecal microbiota transplantation (FMT) for severe dysbiosis, and pharmaceuticals such as metformin and allopurinol, which have been shown to modulate the gut microbiota. Crucially, preventative oral care, including diligent hygiene practices, serves as an essential and accessible intervention to disrupt this pathological loop, particularly for patients with metabolic and autoimmune conditions, thereby mitigating systemic inflammatory burden. Unraveling the complex dynamics of this oral–gut continuum is key to developing precise diagnostic tools and effective treatments that address disease at its source [39,49,50,51,52,53,54].

## 7. Future Directions

Prospective research necessitates the integration of multi-omics strategies, including metagenomics, metabolomics, and immunomics, to systematically delineate the intricate molecular interactions governing the oral–gut–systemic axis. The complexity of these datasets requires advanced bioinformatic platforms and systems biology approaches for effective integration, moving beyond traditional analyses that often fail to capture the holistic interplay between different biological layers. A critical next step is the execution of rigorous, randomized clinical trials to validate the efficacy of microbiome-modulating therapies, such as probiotics or prebiotics, in patients with periodontitis and concurrent systemic comorbidities. For example, a recent study demonstrated that paraprobiotic-based toothpaste and mouthwash, used alongside scaling and root planning, significantly improved clinical periodontal parameters and reduced pathogenic bacterial counts more effectively than conventional chlorhexidine treatment [55]. Such trials are essential for transitioning promising mechanistic findings into clinically actionable interventions. Ultimately, the synthesis of these multi-omics data and clinical evidence will inform the development of precision dentistry, where individualized patient care is guided by systemic health profiles, thereby ushering in a new standard of preventative and therapeutic dental practice. This paradigm shift will leverage biomarker identification and predictive modeling to mitigate disease progression and improve overall patient outcomes in the era of precision medicine

## 8. Conclusions

The oral–gut–systemic axis offers a unifying mechanistic framework linking periodontitis and systemic diseases. The bidirectional relationship between oral and gut dysbiosis underscores the importance of oral health in systemic disease prevention and management. Clinical translation of these insights holds promise for diagnostics, prognostics, and microbiome-targeted therapies

The robust mechanistic framework of the oral–gut–systemic axis demands a paradigm shift from reactive treatment to proactive, integrated healthcare. Multidisciplinary teams, including dental, medical, and nutritional specialists, must collaborate to implement comprehensive prevention and management strategies. By leveraging advanced diagnostics and precision microbiome-targeted therapies, such as specific prebiotics and probiotics, clinicians can actively modulate dysbiosis to prevent the onset and progression of chronic disease. This integrative approach not only improves patient outcomes but also redefines systemic health by placing oral and gut microbial balance at the forefront of holistic care.

## Figures and Tables

**Table 1:** Oral Pathogens, their Mechanisms in the Gut/Immune Axis, and Associated Systemic Effects.

Oral Pathogen	Mechanism in Gut/Immune Axis	Systemic Effect(s)
*Fusobacterium nucleatum*	FadA adhesin (E-cadherin/β-catenin signaling); Fap2 (immune evasion)	Colorectal cancer promotion, colitis exacerbation
*Porphyromonas gingivalis*	Disruption of gut barrier (↓ZO-1, Ocln); bile acid & amino acid metabolism	NAFLD progression, metabolic syndrome, glucose intolerance, RA aggravation, neuroinflammation
*Streptococcus mutans* (CBP+)	Collagen-binding protein → vascular adhesion & platelet disruption	Hemorrhagic stroke, aggravation of ulcerative colitis
*Klebsiella* spp.	Gut colonization → Th1 immune activation	Colitis, systemic immune activation
*Haemophilus parainfluenzae*	Ectopic colonization in gut	Exacerbation of Crohn’s disease/IBD
General oral dysbiosis	Translocation, Th17/Treg imbalance, systemic inflammation	Obesity-related bone loss, atherosclerosis, systemic autoimmunity

## Data Availability

Not applicable.

## Abbreviations

The following abbreviations are used in this manuscript:

*P. gingivalis*	*Porphyromonas gingivalis*
*F. nucleatum*	*Fusobacterium nucleatum*
CRC	Colorectal Cancer
FadA	Fusobacterium adhesin A (protein)
Fap2	Fusobacterium protein involved in immune evasion
TIGIT	T-cell Immunoreceptor with Ig and ITIM domains
IL	Interleukin (e.g., IL-17)
TNF-α	Tumor Necrosis Factor alpha
ZO-1	Zonula Occludens-1 (tight junction protein)
Ocln	Occludin (tight junction protein)
NAFLD	Non-Alcoholic Fatty Liver Disease
RA	Rheumatoid Arthritis
UC	Ulcerative Colitis
CBP	Collagen-Binding Protein
Cnm	Collagen-binding protein of *Streptococcus mutans*
TMAO	Trimethylamine N-oxide
IBD	Inflammatory Bowel Disease
HMA	Human Microbiota-Associated (mouse models)
LPS	Lipopolysaccharide
Th17	T helper 17 cells
Tregs	Regulatory T cells
SCFAs	Short-Chain Fatty Acids
FMT	Fecal Microbiota Transplantation
